# Transcriptional control of hydrogen peroxide homeostasis regulates ground tissue patterning in the *Arabidopsis* root

**DOI:** 10.3389/fpls.2023.1242211

**Published:** 2023-08-21

**Authors:** Jiyeong Oh, Ji Won Choi, Sejeong Jang, Seung Woo Kim, Jung-Ok Heo, Eun Kyung Yoon, Soo-Hwan Kim, Jun Lim

**Affiliations:** ^1^Department of Systems Biotechnology, Konkuk University, Seoul, Republic of Korea; ^2^Division of Biological Science and Technology, Yonsei University, Wonju, Republic of Korea

**Keywords:** *Arabidopsis*, class III peroxidase, gibberellic acid, ground tissue, hydrogen peroxide, middle cortex formation, root development

## Abstract

In multicellular organisms, including higher plants, asymmetric cell divisions (ACDs) play a crucial role in generating distinct cell types. The *Arabidopsis* root ground tissue initially has two layers: endodermis (inside) and cortex (outside). In the mature root, the endodermis undergoes additional ACDs to produce the endodermis itself and the middle cortex (MC), located between the endodermis and the pre-existing cortex. In the *Arabidopsis* root, gibberellic acid (GA) deficiency and hydrogen peroxide (H_2_O_2_) precociously induced more frequent ACDs in the endodermis for MC formation. Thus, these findings suggest that GA and H_2_O_2_ play roles in regulating the timing and extent of MC formation. However, details of the molecular interaction between GA signaling and H_2_O_2_ homeostasis remain elusive. In this study, we identified the *PEROXIDASE 34* (*PRX34*) gene, which encodes a class III peroxidase, as a molecular link to elucidate the interconnected regulatory network involved in H_2_O_2_- and GA-mediated MC formation. Under normal conditions, *prx34* showed a reduced frequency of MC formation, whereas the occurrence of MC in *prx34* was restored to nearly WT levels in the presence of H_2_O_2_. Our results suggest that PRX34 plays a role in H_2_O_2_-mediated MC production. Furthermore, we provide evidence that SCARECROW-LIKE 3 (SCL3) regulates H_2_O_2_ homeostasis by controlling transcription of *PRX34* during root ground tissue maturation. Taken together, our findings provide new insights into how H_2_O_2_ homeostasis is achieved by SCL3 to ensure correct radial tissue patterning in the *Arabidopsis* root.

## Introduction

Plants dynamically integrate environmental signals into their genetic programs to provide flexibility for growth and development. Plant hormones have been shown to play key roles in such signaling pathways (Achard et al., 2006; Wolters and Jürgens, 2009; Verma et al., 2016). Despite the complexity of the pathways, signals are ultimately relayed to transcription factors (TFs) to spatiotemporally regulate cellular behaviors and responses (Srivastava et al., 2010; Moreno-Risueno et al., 2015; Barah et al., 2016). Identifying and characterizing transcriptional regulatory networks are important for understanding the cellular processes involved in plant growth and development (Moreno-Risueno et al., 2015; Chaiwanon et al., 2016; Dhar et al., 2022).

Multicellular organisms such as animals and plants require asymmetric cell divisions (ACDs) to generate distinct cell types during development (Horvitz and Herskowitz, 1992; Knoblich, 2008; Ten Hove and Heidstra, 2008; Abrash and Bergmann, 2009; De Smet and Beeckman, 2011; Smolarkiewicz and Dhonukshe, 2013). Therefore, the timing and extent of ACDs should be precisely controlled to ensure correct cell/tissue patterning. In the *Arabidopsis* (*Arabidopsis thaliana*) root, stem cells of the ground tissue (known as cortex/endodermis initial; CEI) undergo anticlinal ACDs to generate both self-renewed CEI and its daughter cell (known as cortex/endodermis initial daughter; CEID). Subsequent periclinal ACDs of the CEID cells produce two ground tissue layers (endodermis and cortex) from embryogenesis onwards (Benfey et al., 1993; Dolan et al., 1993; Scheres et al., 1994; Scheres et al., 1995; Di Laurenzio et al., 1996; Helariutta et al., 2000; Cui et al., 2007; Cruz-Ramirez et al., 2012). Therefore, in the early developmental phases, the *Arabidopsis* root has an endodermis (inside) and a cortex (outside) in the ground tissue. Around 7 days post-germination (dpg), another round of endodermal ACDs occurs to generate both endodermis and additional cortex (termed middle cortex; MC), resulting in three ground tissue layers: endodermis, MC, and cortex (inside to outside) (Baum et al., 2002; Paquette and Benfey, 2005). Therefore, the onset of endodermal ACDs has been used to assess the maturation of the *Arabidopsis* root ground tissue by measuring the proportion of plants with MC (Baum et al., 2002; Paquette and Benfey, 2005; Cui and Benfey, 2009a; Cui and Benfey, 2009b; Heo et al., 2011; Koizumi et al., 2012a; Koizumi et al., 2012b; Gong et al., 2016; Lee et al., 2016; Bertolotti et al., 2021; Xie et al., 2023).

Accumulating evidence has revealed that developmental pathways and plant hormones interact to modulate the timing and extent of endodermal ACDs for MC formation (Paquette and Benfey, 2005; Cui and Benfey, 2009a; Cui and Benfey, 2009b; Heo et al., 2011; Pauluzzi et al., 2012; Petricka et al., 2012; Koizumi et al., 2012a; Koizumi et al., 2012b; Cui et al., 2014; Choi and Lim, 2016; Cui, 2016; Gong et al., 2016; Lee et al., 2016; Di Ruocco et al., 2018; Bertolotti et al., 2021; Hernández-Coronado and Ortiz-Ramírez, 2021; Shtin et al., 2022; Xie et al., 2023). Previous studies have highlighted the role of GA in controlling MC generation (Paquette and Benfey, 2005; Cui and Benfey, 2009a; Cui and Benfey, 2009b; Heo et al., 2011; Koizumi et al., 2012b; Gong et al., 2016; Lee et al., 2016; Bertolotti et al., 2021). Under GA-deficient conditions caused by the GA biosynthesis inhibitor paclobutrazol (PAC) or the loss-of-function mutation in the key GA biosynthesis gene (e.g., *ga1-3*), the endodermis undergoes more excessive periclinal ACDs for MC production than in the wild-type (WT) (Paquette and Benfey, 2005; Cui and Benfey, 2009a; Cui and Benfey, 2009b; Heo et al., 2011; Koizumi et al., 2012b; Gong et al., 2016; Lee et al., 2016). Interestingly, SCARECROW-LIKE 3 (SCL3), a member of the GRAS transcription factor family, acts as a tissue-specific GA signaling integrator in GA-mediated MC formation (Heo et al., 2011; Gong et al., 2016; Lee et al., 2016). The loss of SCL3 function mutant (*scl3-1*) displayed precocious endodermal ACDs, whereas the overexpression of *SCL3* (*SCL3-OX*) resulted in a reduced frequency of MC formation (Heo et al., 2011; Lee et al., 2016). Therefore, these findings suggest that transcriptional inputs integrated by SCL3 play key roles in GA regulation of endodermal ACDs for MC formation (Heo et al., 2011; Choi and Lim, 2016; Gong et al., 2016; Lee et al., 2016).

Reactive oxygen species (ROS) are inevitably generated as undesirable byproducts in aerobic organisms (Mittler et al., 2004; Livanos et al., 2012). ROS also act as important signaling molecules that control diverse processes in plant growth and development (Apel and Hirt, 2004; Gapper and Dolan, 2006; Tsukagoshi et al., 2010; Xia et al., 2015; Tsukagoshi, 2016; Zhou et al., 2020; Mase and Tsukagoshi, 2021). In particular, H_2_O_2_, a relatively stable type of ROS, regulates MC formation (Cui et al., 2014; Li et al., 2020). For instance, when supplemented with H_2_O_2_, ACDs were more frequently observed in the endodermis (Cui et al., 2014). Conversely, the occurrence of MC was reduced in roots treated with the H_2_O_2_ scavenger, potassium iodide (KI), compared to untreated roots (Li et al., 2020). In addition, previous work implied that a subset of class III peroxidases (PRXs) might play a role in the H_2_O_2_-mediated modulation of MC generation (Cui et al., 2014). Class III PRXs are plant-specific secretory peroxidases, which belong to multigene families with a diverse range of functions (Tognolli et al., 2002; Valério et al., 2004; Dunand et al., 2007; Cosio and Dunand, 2009; Francoz et al., 2015; Shigeto and Tsutsumi, 2016). Apoplastic PRXs have different functions and reactivities that facilitate either ROS generation or scavenging in the context of reactions (Tognolli et al., 2002; Valério et al., 2004; Dunand et al., 2007; Cosio and Dunand, 2009; Francoz et al., 2015; Shigeto and Tsutsumi, 2016). However, their roles in H_2_O_2_-mediated MC formation are currently unknown. Furthermore, the molecular link between H_2_O_2_ and GA-mediated periclinal ACDs in the endodermis remains unclear.

In this study, to better understand H_2_O_2_-mediated endodermal ACDs for MC formation, we identified and characterized a transcriptional regulatory network in ground tissue maturation. Our results have confirmed that H_2_O_2_ generation facilitates MC production in the *Arabidopsis* root. Importantly, we provide convincing evidence that SCL3 plays a role in H_2_O_2_ homeostasis through transcriptional regulation of the *PRX34* gene in H_2_O_2_-mediated MC formation.

## Materials and methods

### Plant material and growth conditions

*Arabidopsis thaliana* ecotype Columbia (Col-0) was used as the WT control. The mutant and transgenic lines used in this study were *pCO2::H_2_B-YFP* (Heidstra et al., 2004; Heo et al., 2011), *ga1-3* (Sun and Kamiya, 1994; Heo et al., 2011; Zhang et al., 2011), *prx34* (SALK_051769) (Passardi et al., 2006; Han et al., 2015), *scl3-1* and *SCL3-OX* (Heo et al., 2011; Zhang et al., 2011). As described previously (Heo et al., 2011; Lee et al., 2016), seeds were surface-sterilized, imbibed at 4°C in the dark, and grown on half-strength of Murashige-Skoog (MS) agar plates (1/2 MS salt mixture, 0.5 mM MES, pH5.7-5.8, 1% sucrose, and 1% agar). To verify homozygous plants from genetic crosses, PCR-based genotyping was performed as previously described (Heo et al., 2011; Lee et al., 2016). For genetic crosses and seed multiplication, seedlings on 1/2 MS agar plates were transferred to soil and grown under long-day conditions (16 h light/8 h dark cycles) as described previously (Heo et al., 2011; Lee et al., 2016). Sequences of the PCR primers used for genotyping are listed in **Supplementary Table S1**.

### MC formation analysis

For phenotypic analysis of MC formation, approximately 36-40 hours post-germination (hpg) seeds were transferred to new 1/2 MS agar plates supplemented with different chemicals, including H_2_O_2_ (100 μM; Sigma-Aldrich, USA), KI (1 mM; Duchefa Biochemie, Netherlands), or PAC (1 µM; Duchefa Biochemie, Netherlands). The same batches of the seeds were transferred to new 1/2 MS agar plates with no supplemented chemicals as controls. More than 300 roots from three replicate experiments (each experiment, n > 100 roots per treatment or genotype) were observed using an Axio Imager.A1 microscope (Carl Zeiss, Germany), and the frequency of endodermal ACDs for MC formation was measured, as previously described (Heo et al., 2011; Lee et al., 2016). Simultaneously, seedlings (n > 30 roots per treatment or genotype) grown on 1/2 MS agar plates with different supplements were observed using a Zeiss LSM 800 confocal laser scanning microscope (Carl Zeiss, Germany), as previously described (Lee et al., 2016; Yoon et al., 2016; Dhar et al., 2022). Student’s *t*-test was performed using Microsoft Excel (Microsoft, USA) and the data presented herein are means values ± standard error (SEM) as described previously (Heo et al., 2011; Lee et al., 2016; Yoon et al., 2016; Dhar et al., 2022).

### Reverse transcription-associated quantitative PCR (RT-qPCR)

Total RNA samples were extracted from WT, mutant, and transgenic seedling roots grown on 1/2 MS agar plates, and used for cDNA synthesis and RT-qPCR as described previously (Heo et al., 2011; Lee et al., 2016; Yoon et al., 2016; Dhar et al., 2022). *ACTIN2* (*ACT2*; AT3G18780) was used as an internal reference (Yoon et al., 2016; Dhar et al., 2022). Each experiment was independently performed with at least three biological replicates, as previously described (Heo et al., 2011; Lee et al., 2016; Yoon et al., 2016; Dhar et al., 2022). Student’s *t*-test was performed using Microsoft Excel (Microsoft, USA).

### H_2_O_2_ assays

Qualitative and quantitative assays were conducted to analyze H_2_O_2_ concentrations in seedling roots. DAB staining (3,3′-diaminobenzidine; Sigma-Aldrich, USA) was used to qualitatively assess H_2_O_2_ concentration, as previously described (Thordal-Christensen et al., 1997; Vanacker et al., 2000; Bindschedler et al., 2006; Daudi et al., 2012) with minor modifications. DAB solution (final concentration: 1 mg/mL) was prepared in 0.05% Triton X-100 (v/v) and 10 mM sodium phosphate buffer. The seedlings were vacuum-infiltrated in the DAB solution for 10 min and subsequently incubated at 30°C for 30 min in the dark. After termination of the staining reaction, samples were fixed in the bleaching solution [ethanol:acetic acid:glycerol, 3:1:1 (v/v/v)] at 95°C for 15 min. Seedling roots were observed using an Axio Imager.A1 microscope equipped with an AxioCam MRc5 digital camera (Carl Zeiss, Germany). The intensity of DAB staining in the seedling roots was quantified by NIH Image J software (http://rsb.info.nih.gov/ij; Schneider et al., 2012), as described previously (Li et al., 2020). To quantitatively measure H_2_O_2_ concentration, we used the Amplex^®^ Red Hydrogen Peroxide Assay Kit (cat. #A22188, Invitrogen), according to the manufacturer’s instructions. The 7 dpg seedling roots grown on 1/2 MS agar plates with or without supplemented chemicals were pulverized in liquid nitrogen. Subsequently, five volumes of 50 mM sodium phosphate buffer (pH 7.4) were added, mixed thoroughly, and incubated on ice for 10 min. The samples were centrifuged at 12,000 rpm for 20 min at 4°C, and supernatants were used for the measurement of H_2_O_2_ concentration as previously described (Cui et al., 2014). Each experiment was independently repeated at least three times and data were analyzed using Microsoft Excel (Microsoft, USA).

## Results

### Induction of H_2_O_2_ by GA deficiency facilitates MC formation

Previously, it has been shown that H_2_O_2_ promotes periclinal ACDs in the endodermis for MC production (Cui et al., 2014; Li et al., 2020). These findings suggest that H_2_O_2_ homeostasis plays a role in modulating the timing and extent of MC formation in the root ground tissue. We thus verified that the frequency of MC occurrence in Columbia wild-type (hereafter referred to as WT) roots was elevated by H_2_O_2_. Under normal growth conditions, cells in the endodermis of 4 dpg WT seedlings barely divided to generate the MC layer, whereas endodermal ACDs were frequently observed under H_2_O_2_ (100 μM) treatment (~3.7% vs. ~10%) (**Supplementary Figures 1A–C**). In the presence of KI (1 mM), an efficient scavenger of H_2_O_2_ (Dunand et al., 2007), MC formation in 7 dpg WT seedlings was attenuated compared to that in untreated roots (~10.7% vs. ~22%) (**Supplementary Figures 1D–F**). Interestingly, the application of H_2_O_2_ in the presence of KI almost restored the frequency of MC formation to that in untreated WT roots (**Supplementary Figure 1F**). Therefore, consistent with previous studies (Cui et al., 2014; Li et al., 2020), our findings strongly support the notion that H_2_O_2_ induces ACDs in the endodermis for MC production in the root ground tissue.

**Figure 1:**
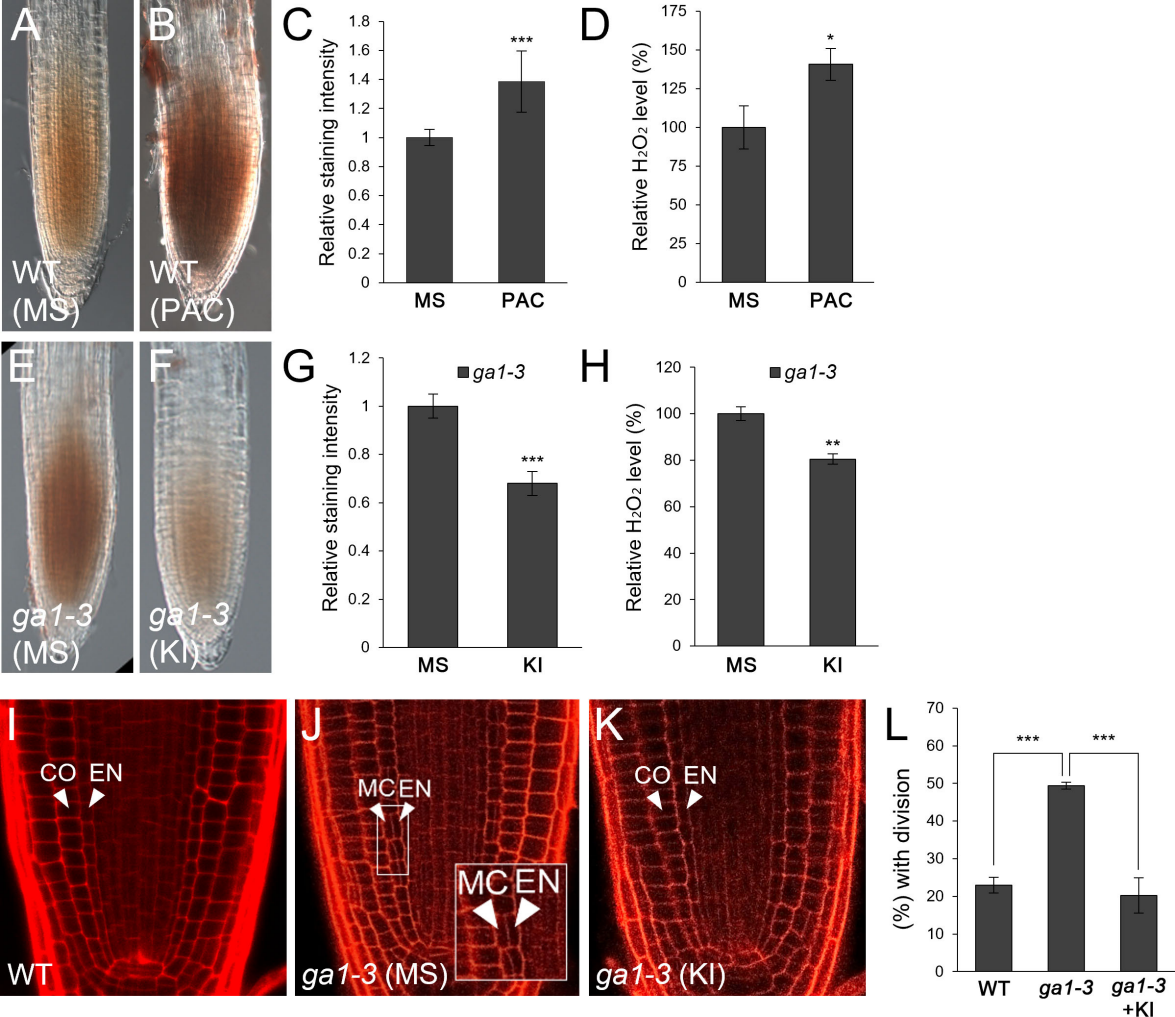
H_2_O_2_ generation by GA deficiency facilitates MC formation in the *Arabidopsis* root. **(A, B)** DAB staining of WT roots in the absence **(A)** or presence **(B)** of PAC. **(C)** Quantification of DAB staining of the WT roots with or without PAC. **(D)** Measurement of H_2_O_2_ in PAC-treated or -untreated WT roots. **(E, F)** DAB staining of *ga1-3* roots in the absence **(E)** or presence **(F)** of KI. **(G)** Quantification of DAB staining of *ga1-3* roots with or without KI. **(H)** Measurement of H_2_O_2_ in KI-treated or -untreated *ga1-3* roots. **(I-K)** Confocal images of WT **(I)** and *ga1-3* roots in the absence **(J)** or presence **(K)** of KI. The inset in **(J)** shows endodermal ACDs for MC formation. The endodermis (EN), middle cortex (MC), and cortex (CO) layers are indicated with white arrowheads. **(L)** Proportion of WT and *ga1-3* plants with MC in the absence or presence of KI. Significance of difference was statistically determined by Student’s *t*-test (*P < 0.05; **P < 0.01; ***P < 0.001).

GA-deficient conditions caused by the loss-of-function mutant *ga1-3* or by the GA biosynthesis inhibitor PAC facilitate endodermal ACDs for the MC layers (Paquette and Benfey, 2005; Cui and Benfey, 2009a; Heo et al., 2011; Koizumi et al., 2012a; Koizumi et al., 2012b; Gong et al., 2016; Lee et al., 2016). Thus, we hypothesized that frequent MC formation might be due to elevated H_2_O_2_ levels induced by GA deficiency. To test this, we first analyzed H_2_O_2_ concentration in roots by DAB staining in the absence or presence of PAC. DAB staining was more intense in PAC-treated WT roots than untreated controls (**Figures 1A–C**). In addition, we quantitatively assessed H_2_O_2_ levels in PAC-treated roots relative to those in untreated controls. Consistent with the DAB staining results, H_2_O_2_ accumulation was higher in PAC-treated roots than untreated controls (**Figure 1D**). Likewise, H_2_O_2_ was more accumulated in *ga1-3* than WT (**Figures 1A, E**). In the presence of KI, *ga1-3* roots showed reduced H_2_O_2_ accumulation (**Figures 1F–H**). These findings indicate that GA-deficient conditions promoted H_2_O_2_ generation. Next, we assessed the frequency of MC formation in *ga1-3* with or without KI. As expected, the occurrence of the MC layers was substantially reduced in KI-treated *ga1-3* compared to that in untreated *ga1-3* (**Figures 1J–L**). Intriguingly, the KI-treated *ga1-3* phenotype was almost indistinguishable from that of the WT control (**Figures 1I, K, L**; **Supplementary Figures 1D, F**). This observation indicates that KI treatment reduced H_2_O_2_ accumulation in *ga1-3*, which resulted in decreased MC formation.

Taken together, our results strongly suggest that the induction of H_2_O_2_ under GA-deficient conditions causes more frequent MC production in the root ground tissue.

### Involvement of PRX34 in H_2_O_2_-mediated MC formation

In an attempt to identify the molecular component(s) underlying the H_2_O_2_-mediated regulation of MC production, we encountered an interesting report that the expression levels of class III peroxidase (*PRX*) genes were significantly changed in the-loss-of-function *spindly* (*spy*) mutants (2-fold enrichment with *P* < 0.05; Cui et al., 2014), which have been shown to play a role in GA signaling (Jacobsen and Olszewski, 1993; Jacobsen et al., 1996; Sun and Gubler, 2004). Of the differentially expressed class III peroxidase (*PRX*) genes (Cui et al., 2014), we focused on *PRX34* because this gene fulfilled our criteria: i) its expression levels were induced by GA-deficient conditions (PAC and *ga1-3*) and H_2_O_2_ (**Supplementary Figures 2A–C**), ii) its expression was enriched in roots (**Supplementary Figure 2D**), and iii) its T-DNA insertion mutant was publicly available (Passardi et al., 2006; Daudi et al., 2012; O’Brien et al., 2012). Therefore, in this study, we aimed to elucidate the role of PRX34 in H_2_O_2_-mediated MC formation.

**Figure 2:**
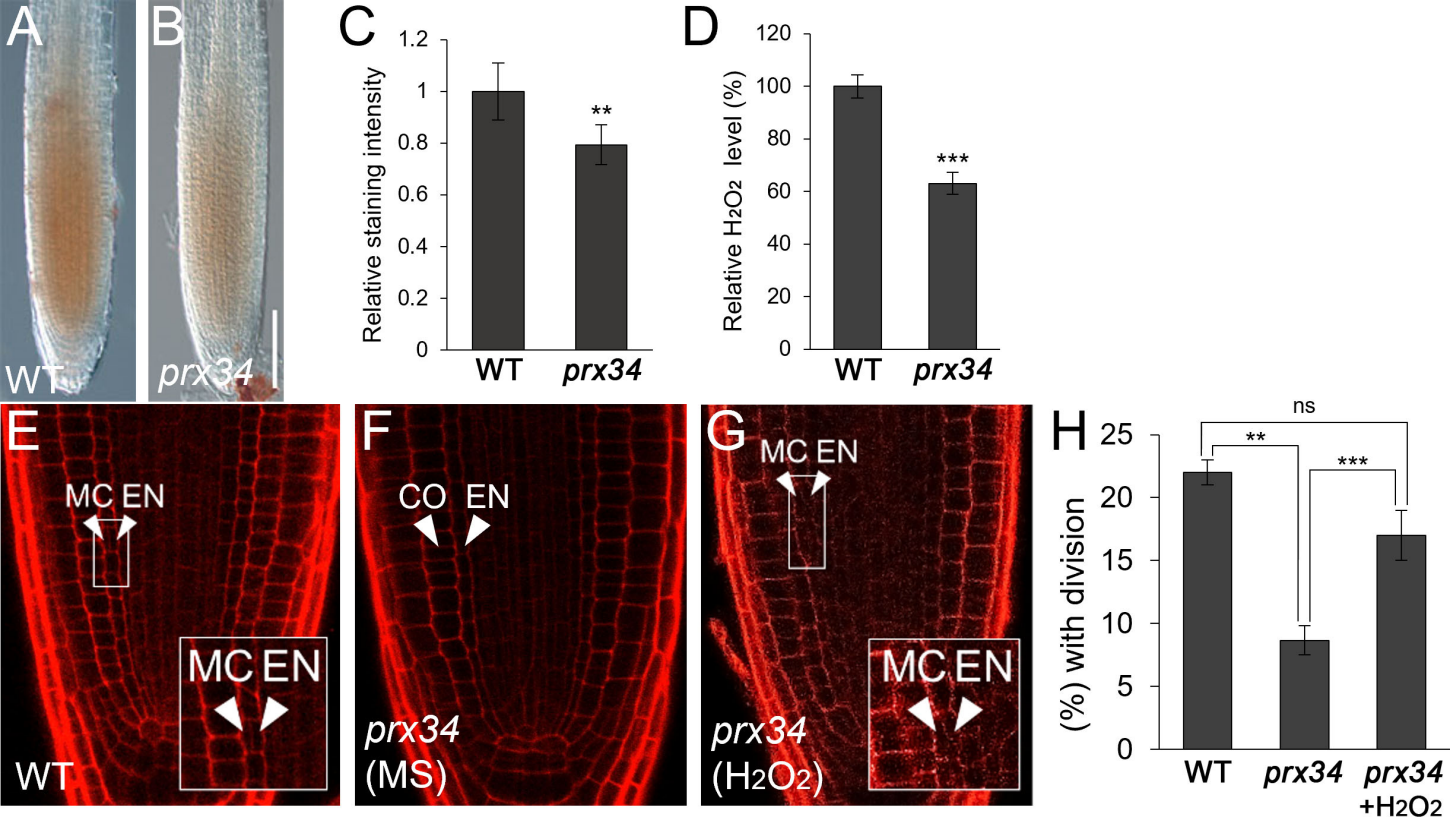
*prx34* roots exhibit reductions of H_2_O_2_ accumulation and MC formation. **(A, B)** DAB staining of WT **(A)** and *prx34* **(B)** roots. **(C)** DAB staining quantification of WT and *prx34* roots. **(D)** Measurement of H_2_O_2_ in WT and *prx34*. **(E-G)** Confocal images of WT **(E)** and *prx34* in the absence **(F)** or presence **(G)** of H_2_O_2_. The insets in **(E)** and **(G)** illustrate periclinal ACDs in the endodermis for MC formation. The endodermis (EN), middle cortex (MC), and cortex (CO) layers are indicated with white arrowheads. **(H)** Proportion of WT and *prx34* plants with MC in the absence or presence of H_2_O_2_. Significance of difference was determined by Student’s *t*-test (**P < 0.01; ***P < 0.001; ns: statistically not significant).

Because PRX34 plays a role in H_2_O_2_ homeostasis (Daudi et al., 2012; O’Brien et al., 2012), we first investigated whether H_2_O_2_ levels were altered in the-loss-of-function *prx34* mutant. Indeed, accumulation of H_2_O_2_ decreased in *prx34* compared to that in WT roots (**Figures 2A–D**). Next, we analyzed the frequency of endodermal ACDs in WT and *prx34* roots. Under normal conditions, *prx34* showed a reduced frequency of MC formation compared to WT (**Figures 2E, F, H**). In the presence of H_2_O_2_, the occurrence of endodermal ACDs for MC generation in *prx34* was almost restored to that in WT (**Figures 2E, G, H**).

Taken together, our findings indicate that PRX34 likely plays a role in H_2_O_2_-mediated MC production in the root ground tissue.

### Involvement of PRX34 in the GA-mediated regulation of MC formation

**Figure 3:**
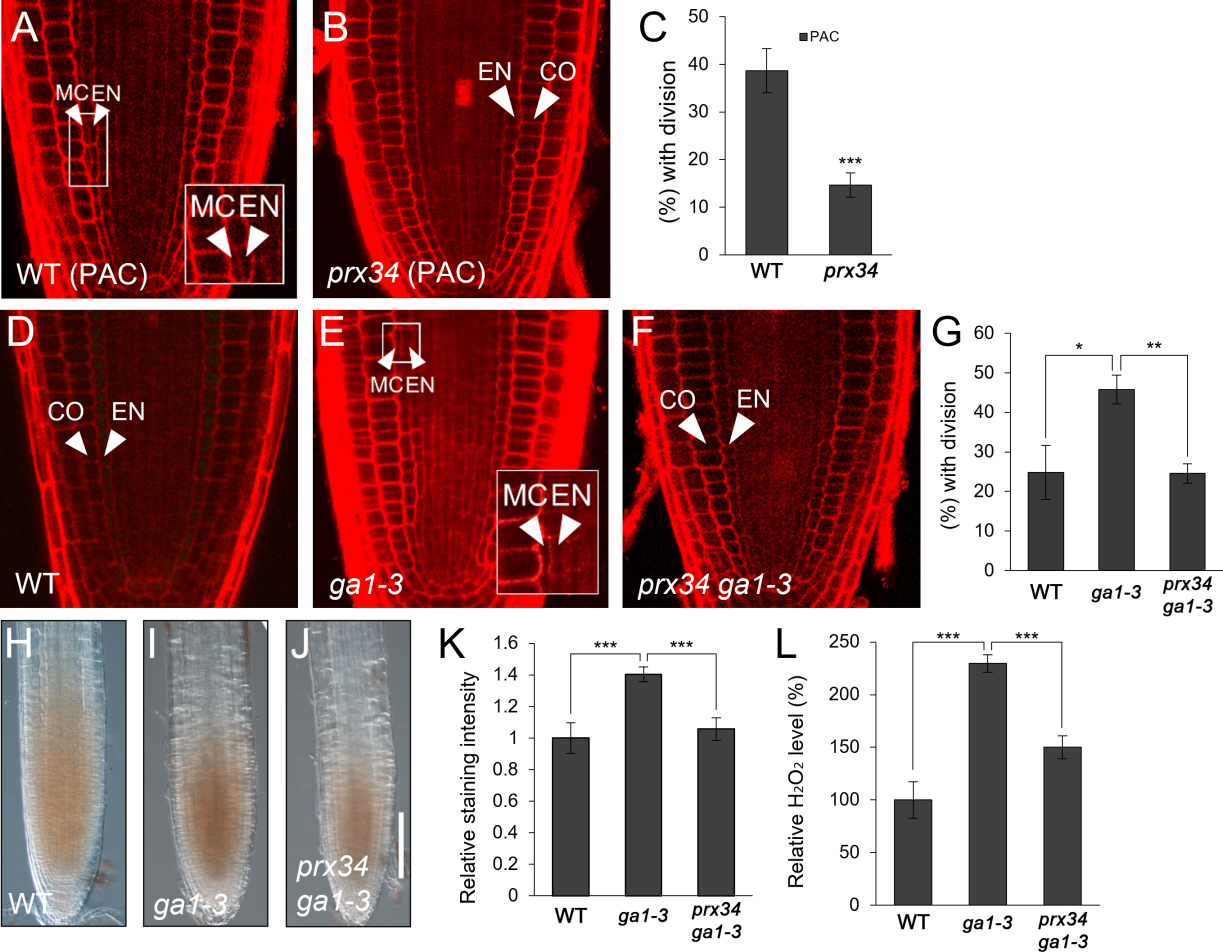
*prx34* roots exhibit reductions of H_2_O_2_ accumulation and MC formation under GA-deficient conditions. **(A, B)** Confocal images of WT **(A)** and *prx34* **(B)** in the presence of PAC. **(C)** Proportion of WT and *prx34* plants with MC in the presence of PAC. **(D-F)** Confocal images of WT **(D)**, *ga1-3* **(E)** and *prx34 ga1-3* **(F)**. The insets in **(A)** and **(E)** illustrate endodermal ACDs for MC formation. The endodermis (EN), middle cortex (MC), and cortex (CO) layers are indicated with white arrowheads. **(G)** Proportion of WT, *ga1-3* and *prx34 ga1-3* plants with the MC layers. **(H-J)** DAB staining of WT **(H)**, *ga1-3* **(I)** and *prx34 ga1-3* **(J)**. **(K)** DAB staining quantification of WT, *ga1-3* and *prx34 ga1-3* roots. **(L)** Measurement of H_2_O_2_ in WT, *ga1-3* and *prx34 ga1-3*. Significance of difference was determined using Student’s *t*-test (*P < 0.05; **P < 0.01; ***P < 0.001).

Considering the results that i) GA deficiency induced H_2_O_2_ accumulation; ii) *PRX34* expression was promoted by GA deficiency and H_2_O_2_; and iii) both H_2_O_2_ level and MC occurrence were substantially attenuated in *prx34*, we hypothesized that PRX34 might be involved in GA-mediated MC formation in the root ground tissue. To test this, we assessed MC production in *prx34* under GA-deficient conditions. In the presence of PAC, *prx34* exhibited a reduced occurrence of the MC layers compared to the WT (**Figures 3A–C**). Next, we performed a genetic analysis using *prx34 ga1-3* double mutants. Compared with *ga1-3* single mutants, *prx34 ga1-3* displayed attenuated MC formation, which was similar to WT (**Figures 3D–G**). To investigate whether the less frequent MC layers in *prx34* under GA-deficient conditions (PAC or *ga1-3*) was due to lower H_2_O_2_ production, we measured the level of H_2_O_2_ in *prx34 ga1-3* roots. The accumulation of H_2_O_2_ in *prx34 ga1-3* was indeed lower than that in *ga1-3* (**Figures 3H–L**). These findings are consistent with our phenotypic analyses in *prx34* roots under GA-deficient conditions.

Taken together, our results suggest that PRX34, via H_2_O_2_ production, is involved in the GA-mediated regulation of MC formation.

### Involvement of SCL3 in H_2_O_2_-mediated MC formation

**Figure 4:**
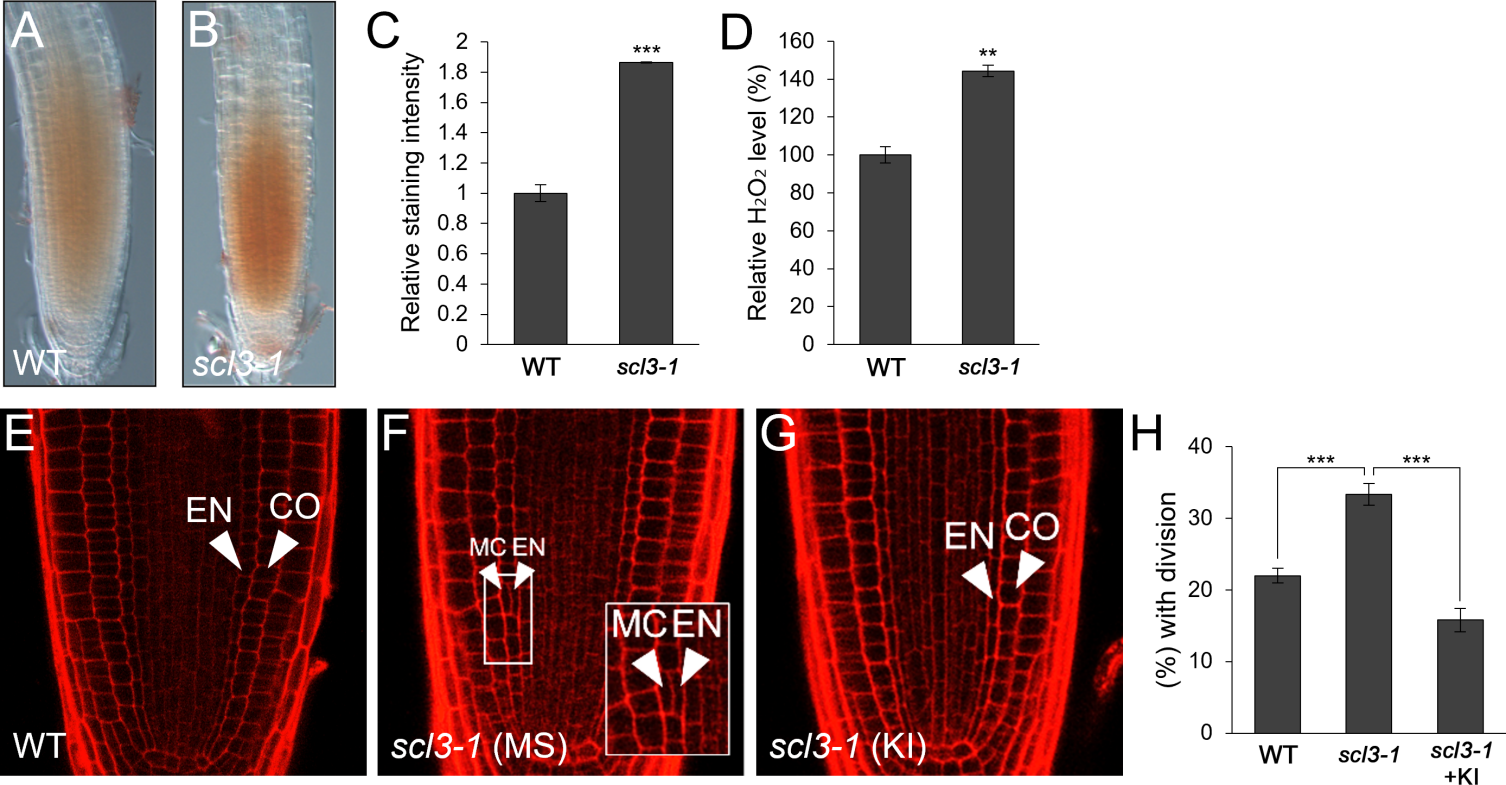
*scl3* plays a role in the regulation of H_2_O_2_-mediated MC formation. **(A, B)** DAB staining of WT **(A)** and *scl3-1* **(B)** roots. **(C)** Quantification of DAB staining in WT and *scl3-1*. **(D)** Measurement of H_2_O_2_ in WT and *scl3-1*. **(E-G)** Confocal images of WT **(E)** and *scl3-1* roots in the absence **(F)** or presence **(G)** of KI. The inset in **(F)** illustrates periclinal ACDs in the endodermis for MC formation. The endodermis (EN), middle cortex (MC), and cortex (CO) layers are indicated with white arrowheads. **(H)** Proportion of WT and *scl3-1* plants with MC in the absence or presence of KI. Significance of difference was determined by Student’s *t*-test (**P < 0.01; ***P < 0.001).

Previously, it has been demonstrated that SCL3 plays a role in GA-mediated MC production and thus, the loss-of-function *scl3-1* results in more frequent MC formation than in WT roots (Heo et al., 2011; Zhang et al., 2011; Lee et al., 2016). Therefore, we hypothesized that the frequent MC generation phenotype in *scl3-1* roots might be due to the elevated H_2_O_2_ level. To test this, we assessed H_2_O_2_ concentration in *scl3-1* in comparison with WT roots and found that *scl3-1* accumulated more H_2_O_2_ than the WT (**Figures 4A–D**). We then analyzed the occurrence of MC in the presence of KI. The frequency of MC formation was substantially reduced in KI-treated *scl3-1* roots compared to untreated controls (**Figures 4E–H**). These findings indicate that increased H_2_O_2_ levels are likely a causative factor in the frequent generation of MC in *scl3-1* roots.

### SCL3 acts upstream of *PRX34* in H_2_O_2_-mediated MC formation

**Figure 5:**
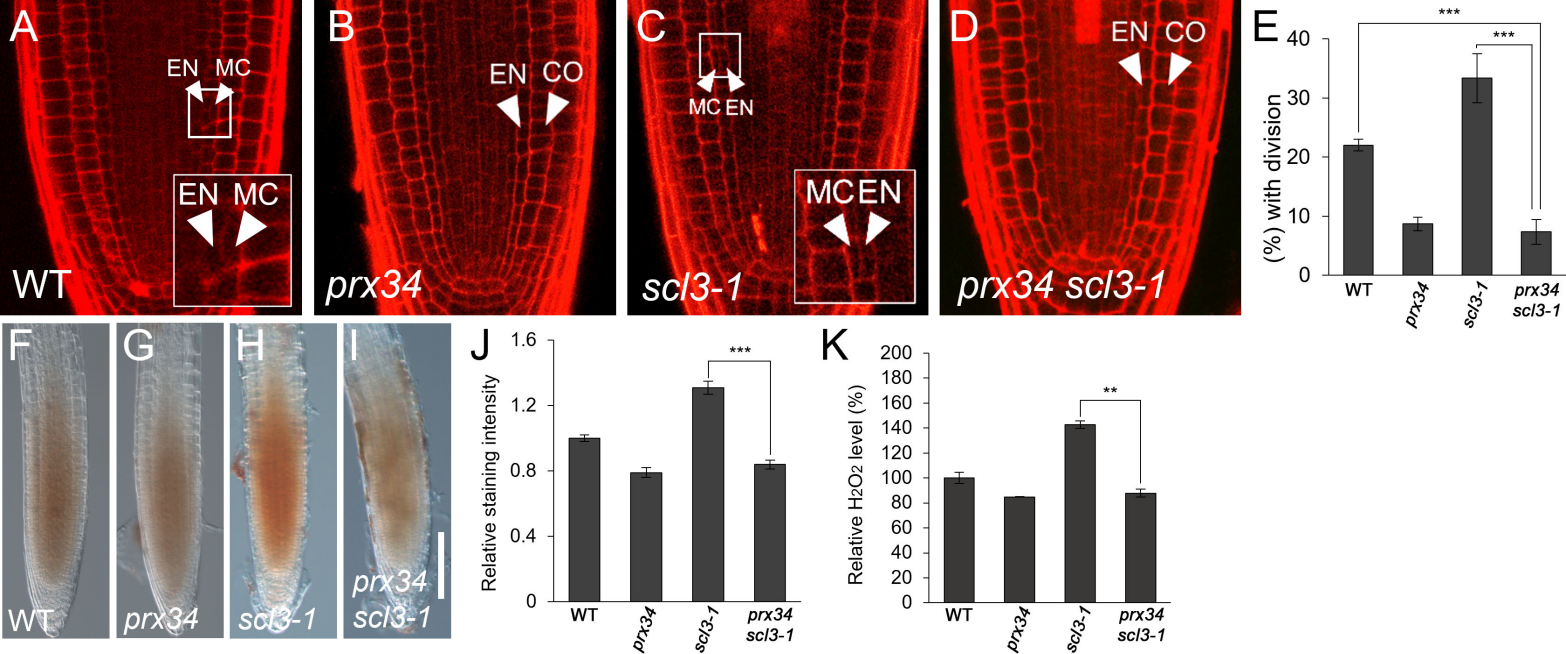
*scl3* roots exhibit reductions of H_2_O_2_ accumulation and MC formation in the loss of PRX34 function. **(A-D)** Confocal images of WT **(A)**, *prx34* **(B)**, *scl3-1* **(C)** and *prx34 scl3-1* **(D)** roots. The insets in **(A)** and **(C)** illustrate endodermal ACDs for MC formation. The endodermis (EN), middle cortex (MC), and cortex (CO) layers are indicated with white arrowheads. **(E)** Proportion of WT, *prx34*, *scl3-1* and *prx34 scl3-1* plants with the MC layers. **(F-I)**. DAB staining of WT **(F)**, *prx34* **(G)**, *scl3-1* **(H)** and *prx34 scl3-1* **(I)** roots. **(J)** Quantification of DAB staining in WT, *prx34*, *scl3-1* and *prx34 scl3-1*. **(K)** Measurement of H_2_O_2_ in WT, *prx34*, *scl3-1* and *prx34 scl3-1*. Significance of difference was determined by Student’s *t*-test (**P < 0.01; ***P < 0.001).

Because SCL3 is likely involved in H_2_O_2_-mediated MC production, we investigated the relationship between SCL3 and PRX34. First, we performed a genetic analysis by generating *prx34 scl3-1* double mutants. Under normal conditions, *prx34* and *scl3-1* single mutants showed opposite phenotypes in MC generation: a decreased occurrence in *prx34* and an increase in *scl3-1* (**Figures 5A–C, E**). Unexpectedly, the MC phenotype of *prx34 scl3-1* double mutants resembled that of *prx34* single mutants; in that the frequency of MC formation was significantly attenuated (**Figures 5B, D, E**). Therefore, our genetic analysis indicates that *prx34* is epistatic to *scl3*. Next, we assessed H_2_O_2_ levels in *prx34 scl3-1* and demonstrated that H_2_O_2_ accumulation in *prx34 scl3-1* was reduced to a level similar to *prx34* (**Figures 5F–K**).

**Figure 6:**
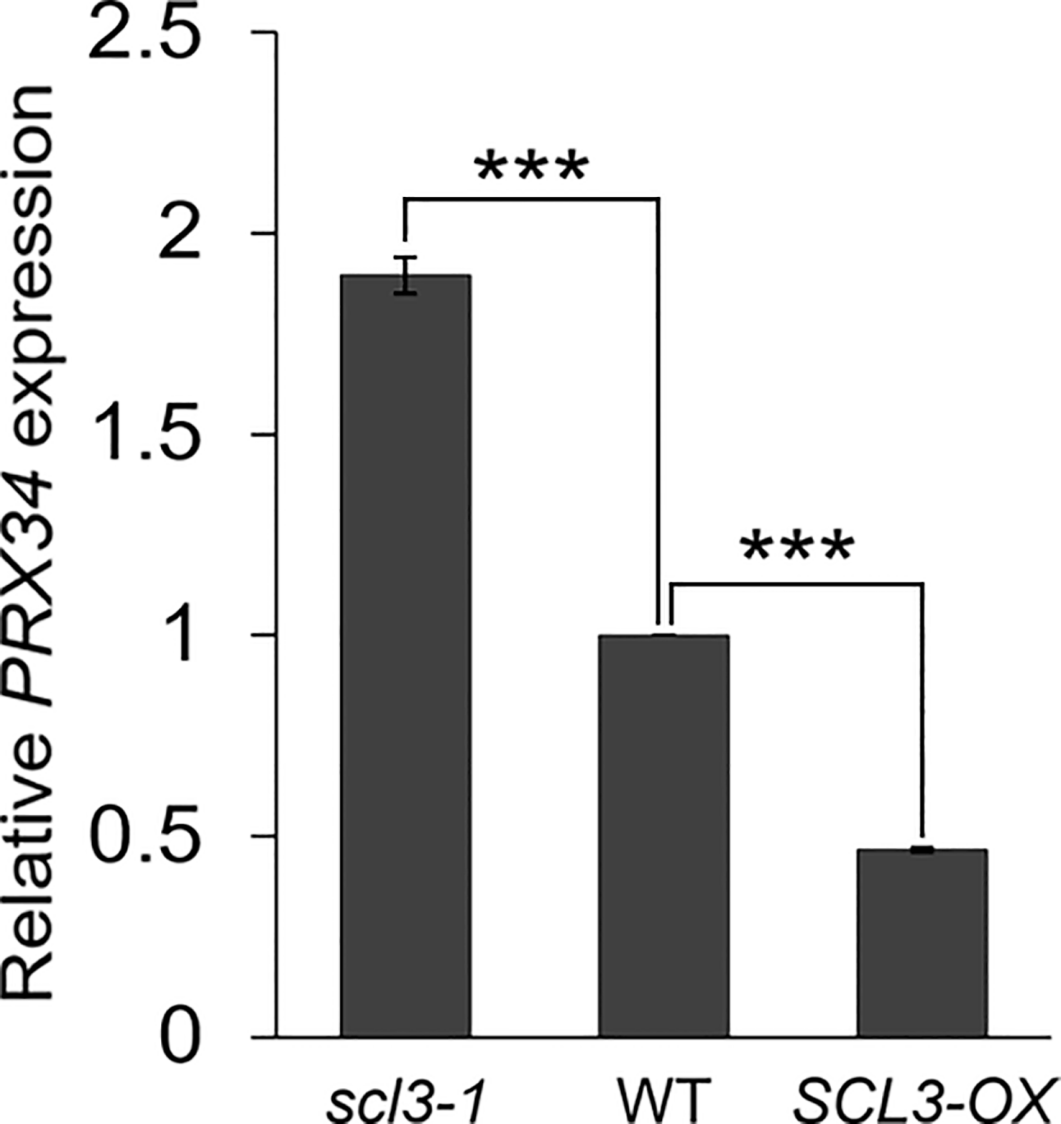
SCL3 negatively regulates the expression levels of *PRX34* in roots. The *PRX34* expression is upregulated in *scl3-1*, whereas its expression is attenuated in *SCL3-OX*. Error bars indicate ± SEM from three independent replicates. Significance of difference was determined by Student’s *t*-test (***P < 0.001).

Our results strongly support the notion that the reduced production of MC in *prx34 scl3-1* is due to the attenuated levels of H_2_O_2_ in the double mutant roots. Furthermore, because *prx34* is epistatic to *scl3*, PRX34 is likely to act downstream of SCL3 in the H_2_O_2_-mediated pathway for MC formation in the root ground tissue. To test this, we analyzed the expression levels of *PRX34* in the loss (*scl3-1*) and gain (*SCL3-OX*) of SCL3 function plants. Interestingly, the abundance of *PRX34* mRNA was elevated in *scl3-1*, whereas its level was reduced in *SCL3-OX* (**Figure 6**). This finding indicates that SCL3 likely acts as a negative regulator to modulate the *PRX34* expression, thereby maintaining H_2_O_2_ homeostasis.

## Discussion

Previously, it has been reported that ROS, particularly H_2_O_2_, induce periclinal ACDs in the endodermis for MC formation in the *Arabidopsis* root (Cui et al., 2014; Li et al., 2020). In agreement with previous reports, we also found that H_2_O_2_ facilitated the occurrence of MC. In addition, the plant hormone GA has been shown to regulate the timing and extent of MC formation (Paquette and Benfey, 2005; Cui and Benfey, 2009a; Cui and Benfey, 2009b; Heo et al., 2011; Pauluzzi et al., 2012; Koizumi et al., 2012a; Koizumi et al., 2012b; Cui et al., 2014; Choi and Lim, 2016; Cui, 2016; Gong et al., 2016; Lee et al., 2016; Bertolotti et al., 2021). Here, we demonstrated that H_2_O_2_ accumulated more in *ga1-3* roots than in WT, resulting in excessive endodermal ACDs for MC production. However, the molecular link between the GA pathway and H_2_O_2_ homeostasis during *Arabidopsis* ground tissue maturation remains elusive.

To better understand the molecular events in H_2_O_2_- and GA-mediated MC formation, we attempted to identify a candidate molecular link using three criteria: i) expression in both GA deficiency and H_2_O_2_, ii) enrichment of expression in the root, and iii) availability of a T-DNA insertion mutant. Previously, it was demonstrated that SPY regulated expression of some class III *PRX* genes (Cui et al., 2014). In particular, the expression levels of *PRX33* and *PRX34*, which are closely related and tandemly located in the *Arabidopsis* genome, were substantially reduced in the *spy* root. Both *PRX33* and *PRX34* were shown to generate H_2_O_2_, which conferred resistance to pathogens during the *Arabidopsis* defense response (Daudi et al., 2012; O’Brien et al., 2012). In addition, both PRXs were reported to be involved in root elongation, possibly modifying cell walls (Passardi et al., 2006). However, the roles of these PRXs in H_2_O_2_-mediated MC formation remain unknown. In this study, we focused our efforts on *PRX34*, which fulfilled our criteria; further investigation of other *PRX* genes is the subject of another study, which is not discussed here. Interestingly, *prx34* roots exhibited a reduction of H_2_O_2_ accumulation, resulting in less frequent endodermal ACDs for MC formation than in WT roots. When applied with H_2_O_2_, the frequency of MC production in *prx34* was restored to WT levels. Furthermore, *prx34* showed reduced MC production in GA-deficient conditions caused by *ga1-3* or PAC, compared to the mutant under normal conditions. These findings strongly support the idea that GA deficiency induces H_2_O_2_ generation via PRX34, and, in turn, H_2_O_2_ accumulation promotes endodermal ACDs for MC formation during *Arabidopsis* ground tissue maturation.

SHORT-ROOT (SHR) and SCARECROW (SCR) play key roles in MC formation (Paquette and Benfey, 2005; Cui and Benfey, 2009a; Cui and Benfey, 2009b; Heo et al., 2011; Koizumi et al., 2012a; Koizumi et al., 2012b; Gong et al., 2016). The *shr* mutant has no endodermis or MC, whereas *scr* exhibits excessive MC production (Paquette and Benfey, 2005; Cui and Benfey, 2009a; Cui and Benfey, 2009b; Heo et al., 2011; Koizumi et al., 2012a; Koizumi et al., 2012b; Gong et al., 2016). Both SHR and SCR directly regulate the expression of the cell cycle regulator *CYCLIND6;1* (*CYCD6;1*) in CEID cells, giving rise to the endodermis and cortex in the root (Sozzani et al., 2010; Cruz-Ramirez et al., 2012). During ground tissue maturation, *CYCD6;1* is upregulated in the endodermis, triggering periclinal ACDs for MC formation (Koizumi et al., 2012a; Koizumi et al., 2012b; Gong et al., 2016; Lee et al., 2016). Recently, it has been reported that SHR promotes H_2_O_2_ accumulation in the *Arabidopsis* root by transcriptionally activating the *RESPIRATORY BURST OXIDASE HOMOLOG* (*RBOH*) genes, which encode NADPH oxidases (Li et al., 2020). In particular, seedling roots ectopically expressing an inducible version of *SHR* (*pG1090-XVE::SHR*; Yu et al., 2017) exhibited increased H_2_O_2_ accumulation and *CYCD6;1* activity, resulting in the frequent occurrence of MC (Li et al., 2020). Therefore, this study suggests that SHR, acting as a positive regulator, plays a role in H_2_O_2_ generation via the transcriptional regulation of the *RBOH* genes.

In the GA signaling pathway, SCL3 antagonizes the function of DELLA proteins, which are the major negative regulators (Silverstone et al., 1997; Silverstone et al., 1998; Sun and Gubler, 2004; Zentella et al., 2007; Heo et al., 2011; Zhang et al., 2011; Yoshida and Ueguchi-Tanaka, 2014; Weng et al., 2020; Ito and Fukazawa, 2021). The *scl3-1* mutant in the *ga1-3* background showed enhanced GA-deficient phenotypes in growth and development, whereas loss-of-function mutations in the *DELLA* genes restored the *ga1-3* phenotypes (Silverstone et al., 1997; Silverstone et al., 1998; Dill and Sun, 2001; Heo et al., 2011; Zhang et al., 2011; Yoshida and Ueguchi-Tanaka, 2014; Weng et al., 2020; Ito and Fukazawa, 2021). In particular, GA-deficient conditions (*ga1-3* or PAC) exacerbate the MC phenotype of *scl3-1*, resulting in excessive endodermal ACDs for MC formation (Heo et al., 2011). When overexpressed, *SCL3-OX* reduced the occurrence of MC, even under GA-deficient conditions (Heo et al., 2011). Thus, SCL3 plays a crucial role in GA-mediated MC formation (Heo et al., 2011). In this study, we demonstrated that H_2_O_2_ accumulation was higher in *scl3-1* than WT. In the presence of KI, the frequency of MC formation in *scl3-1* was attenuated. Furthermore, the *prx34 scl3-1* double mutant showed lower H_2_O_2_ accumulation than *scl3-1*. Consistent with the low level of H_2_O_2_ in *prx34 scl3-1*, the double mutant displayed less frequent MC production than *scl3-1*. Taken together, the *prx34 scl3-1* double mutant was indistinguishable from the *prx34* single mutant in both H_2_O_2_ accumulation and MC phenotype. Thus, our genetic analysis led us to the conclusion that *prx34* is epistatic to *scl3*. In addition, the abundance of *PRX34* transcripts was higher in *scl3-1* and lower in *SCL3-OX* than that in WT. These results strongly suggest that SCL3 serves as a negative regulator of *PRX34* expression to maintain H_2_O_2_ homeostasis during root ground tissue maturation.

SCL3, acting downstream of the SHR/SCR regulatory module, is uniquely positioned in controlling the timing and extent of MC formation (Heo et al., 2011; Choi and Lim, 2016; Gong et al., 2016; Lee et al., 2016). Recently, it has been reported that the SHR/SCR module physically interacted with NAC1 to restrict excessive periclinal ACDs in the endodermis during root ground tissue maturation (Xie et al., 2023). NAC1 directly inhibited the transcription of *CYCD6;1* with the transcriptional co-repressor TOPLESS (TPL), resulting in reduced MC generation (Xie et al., 2023). Thus, it is tempting to speculate that the SHR/SCR regulatory module, together with NAC1, is also involved in H_2_O_2_- and GA-mediated MC formation, impinging on the transcriptional regulation of *SCL3*.

The physiological function of MC formation remains elusive. Except for the *Arabidopsis* root, most plant roots have multiple cortex layers (Esau, 1953; Esau, 1977; Benfey et al., 1993; Dolan et al., 1993; Scheres et al., 1994; Scheres et al., 1995; Di Laurenzio et al., 1996; Helariutta et al., 2000; Cui et al., 2007; Cruz-Ramirez et al., 2012; Wu et al., 2014; Choi and Lim, 2016). However, as the individual *Arabidopsis* plant grows, periclinal ACDs in the endodermis produce an additional cortex layer, namely MC, in the root ground tissue. (Baum et al., 2002; Paquette and Benfey, 2005; Cui and Benfey, 2009a; Cui and Benfey, 2009b; Heo et al., 2011; Pauluzzi et al., 2012; Koizumi et al., 2012a; Koizumi et al., 2012b; Cui et al., 2014; Choi and Lim, 2016; Cui, 2016; Gong et al., 2016; Lee et al., 2016; Di Ruocco et al., 2018; Bertolotti et al., 2021; Hernández-Coronado and Ortiz-Ramírez, 2021; Shtin et al., 2022; Xie et al., 2023). Our results, together with those of previous studies, imply that MC formation caused by GA deficiency and H_2_O_2_ might be a consequence of plant adaptation to environmental stimuli, such as stresses (Cui et al., 2014; Cui, 2015; Choi and Lim, 2016).

**Figure 7:**
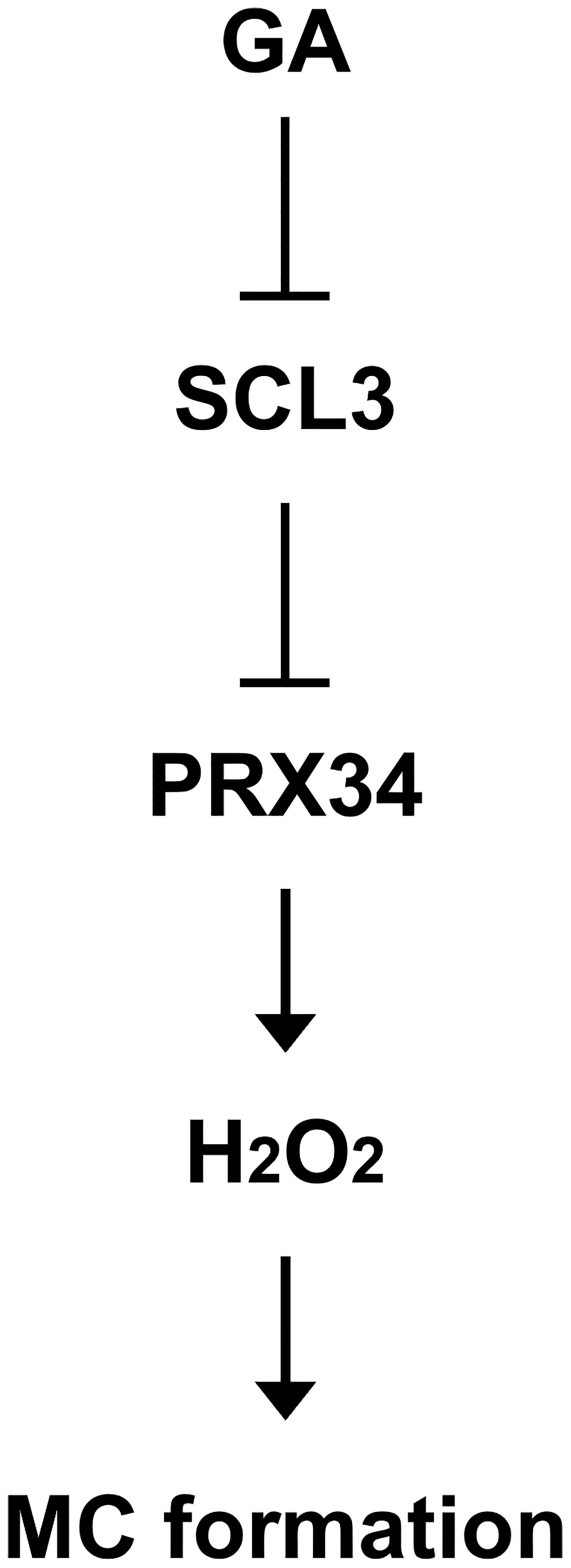
Schematic model for the transcriptional regulation of H_2_O_2_-mediated MC formation in the *Arabidopsis* root. The SCL3 transcription factor, whose expression is modulated by GA, negatively regulates transcription of *PRX34* to maintain H_2_O_2_ homeostasis. The class III peroxidase PRX34, which is positioned downstream of SCL3, produces H_2_O_2_. In turn, H_2_O_2_ accumulation induced by PRX34 promotes periclinal ACDs in the endodermis for MC formation during root ground tissue maturation.

Taken together, in a simplified model (**Figure 7**), the plant hormone GA negatively regulates the expression of *SCL3*. Hence, SCL3 acts as a convergent point for the interaction between GA and ROS. SCL3 plays a role in H_2_O_2_ homeostasis via the transcriptional regulation of *PRX34* in H_2_O_2_-mediated MC formation. Therefore, we have not only provided new insights into the crosstalk between GA and ROS but also unveiled a novel role for *PRX34* in the maintenance of H_2_O_2_ homeostasis during root ground tissue maturation. Thus, it is tempting to speculate that diverse transcriptional inputs from hormonal (e.g., GA) and developmental (e.g., SHR/SCR) pathways impinge on the tissue-specific integrator SCL3 to modulate the timing and extent of MC formation during root ground tissue maturation.

## Data availability statement

The raw data supporting the conclusions of this article will be made available by the authors, without undue reservation.

## Author contributions

JO, JWC, SJ and JL conceived, designed, and performed the experiments. SWK, J-OH, and EKY analyzed the data and performed plant work including genotyping. S-HK contributed new analytical tools and reagents and provided critical comments and suggestions on the experiments. JO, JWC, SJ, and JL wrote the manuscript with contributions from all the authors. All authors contributed to the article and approved the submitted version.
