# Supplementary material for: Transcriptional control of hydrogen peroxide homeostasis regulates ground tissue patterning in the Arabidopsis root
Source: Front Plant Sci. 2023 Aug 21;14:1242211. doi: 10.3389/fpls.2023.1242211 (PMC10475948; doi:10.3389/fpls.2023.1242211)
Supplement: Supplementary file 1 [file DataSheet_1.pdf]

Supplementary Material

Oh et al. (2023). Transcriptional Control of Hydrogen Peroxide Homeostasis Regulates Ground Tissue Patterning in the *Arabidopsis* Root

This PDF includes:

Supplementary Figures: Figure S1 and S2

Supplementary Table: Table S1

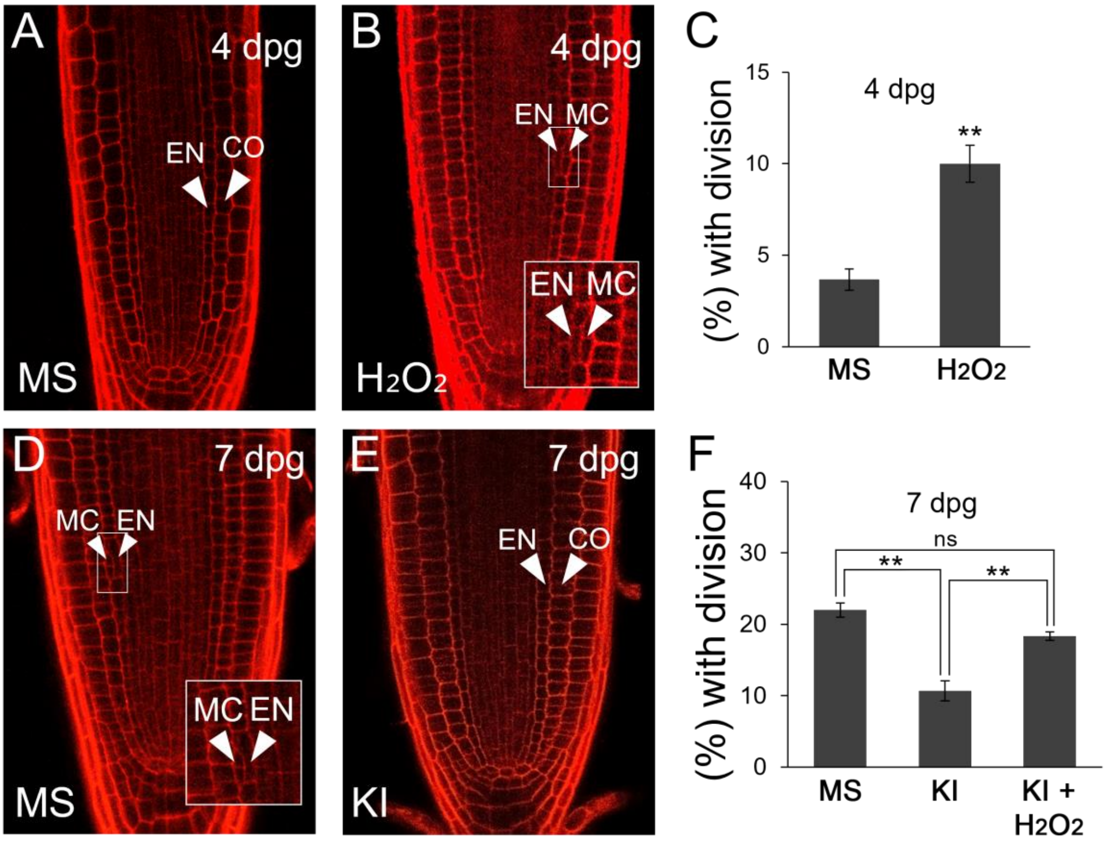

FIGURE S1. H_2O_2 promotes MC formation in the *Arabidopsis* root. **(A,B)** Confocal images of WT roots in the absence **(A)** or presence **(B)** of H_2O_2. The inset in **(B)** shows endodermal ACDs for MC formation. **(C)** Proportion of WT plants with MC in the absence or presence of H_2O_2. **(D,E)** Confocal images of WT roots in the absence **(D)** or presence **(E)** of KI. The insets in **(B,D)** show endodermal ACDs for MC formation. The endodermis (EN), middle cortex (MC), and cortex (CO) layers are indicated with white arrowheads. **(F)** Proportion of WT plants with the MC layers in the absence or presence of KI. Significance of difference was statistically determined by Student's *t*-test (** $P < 0.01$).

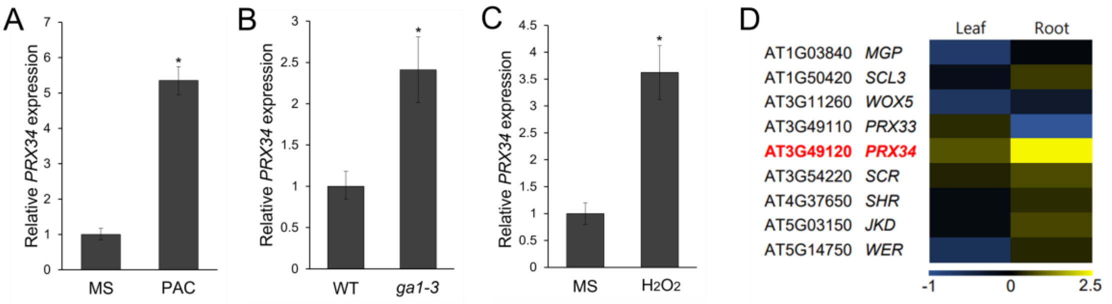

FIGURE S2. Regulation of *PRX34* mRNA abundance by GA deficiency and H_2O_2. **(A,B)** Expression of *PRX34* in WT roots under GA-deficient conditions caused by PAC **(A)** or *ga1-3* **(B)**. Abundance of *PRX34* mRNA in the absence or presence of H_2O_2. **(D)** The heatmap of the *PRX34* expression levels by transcriptome analysis from the public database (https://www.ebi.ac.uk/gxa/home). Significance of difference was statistically determined by Student's *t*-test (*$P < 0.05$).

Table S1. Sequence information for primers.

Purpose	Name		Sequence (5'→3')	Reference
Genotyping	*prx34*	F	CCAATTATCTTTGTTTCAGAC	This study
		R	GGGGTGAGTTGAGCAGCGGAC	
	ga1-3	wt F	TTTGGCCCAACACACAAACAAACCTT	(Heo et al., 2011)
		wt R	AAGCTTCGAACTCAAGGTTCTA	
		mt F	TGTATGCACGTTAACGATCAAT	
		mt R	TTTCTTCATACCACCTGCGTTC	
	scl3-1	F	CACCATGGTGGCTATGTTTCAAGAAG	
		R	TCACTTCCTGCATCTCCAAGC	
	T-DNA	SALK	GGCAATCAGCTGTTGCCCGTCTCACTGGTG	http://signal.salk.edu
RT-qPCR	*PRX34*	F	CTCTTCGTCTTCAACATCGTCC	This study
		R	AGGACATGACCTATCGTAG	
	ACTIN2	F	TCGCTGACCGTATGAGCAAAGAA	(Yoon et al., 2016)
		R	TGGAATGTGCTGAGGGAAGCA	
